# Supplementary material for: Chemotherapeutic Drugs Inhibiting Topoisomerase 1 Activity Impede Cytokine-Induced and NF-κB p65-Regulated Gene Expression
Source: Cancers (Basel). 2019 Jun 25;11(6):883. doi: 10.3390/cancers11060883 (PMC6627772; doi:10.3390/cancers11060883)
Supplement: Supplementary file 1 [file cancers-11-00883-s001.pdf]

Supplementary Materials

Chemotherapeutic Drugs Inhibiting Topoisomerase 1 Activity Impede Cytokine-Induced and NF-κB p65-Regulated Gene Expression

Tabea Riedlinger, Marek Bartkuhn, Tobias Zimmermann, Sandra B. Hake, Andrea Nist, Thorsten Stiewe, Michael Kracht and M. Lienhard Schmitz

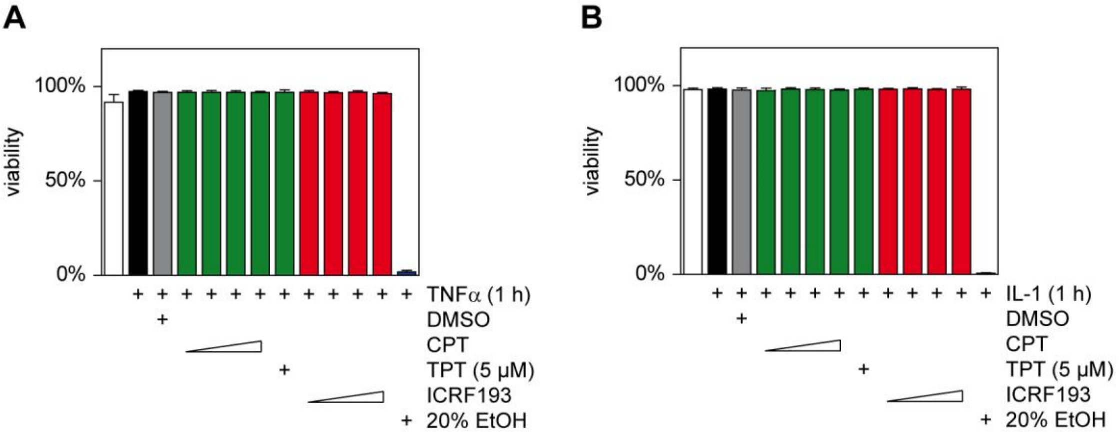

Figure S1. Determination of the effects of TOP inhibitors on HCT116 and KB cell viability. (**A**) HCT116 cells were pre-treated for 2 h with increasing concentrations (0.5 µM, 1 µM, 5 µM, 10 µM) of TOP1 and TOP2 inhibitors and TNFα was added for 1 h as shown, treatment with 20% ethanol was used as a positive control. Attached and floating cells were harvested and washed once in PBS. Using the eBioscienceTM Annexin V Apoptosis Detection Kit FITC (Invitrogen), pellets were then resuspended according to manufacturer's instructions in 1× Binding Buffer. Cell viability was then analyzed via propidium iodide (PI) staining. The percentage of PI negative cells (=viable cells) is shown, SEMs were obtained from three individual experiments. (**B**) The same experiment was performed as described in (**A**) with KB cells, the stimulus was changed for IL-1. Depicted are mean values from three individual experiments, error bars show SEM.

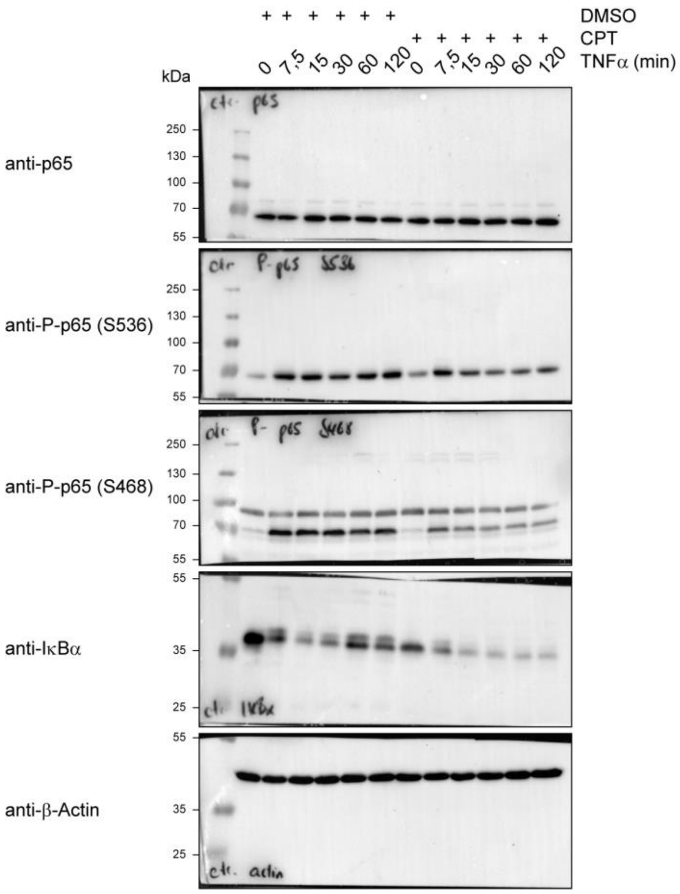

Figure S2. Blots from Figure 1F.

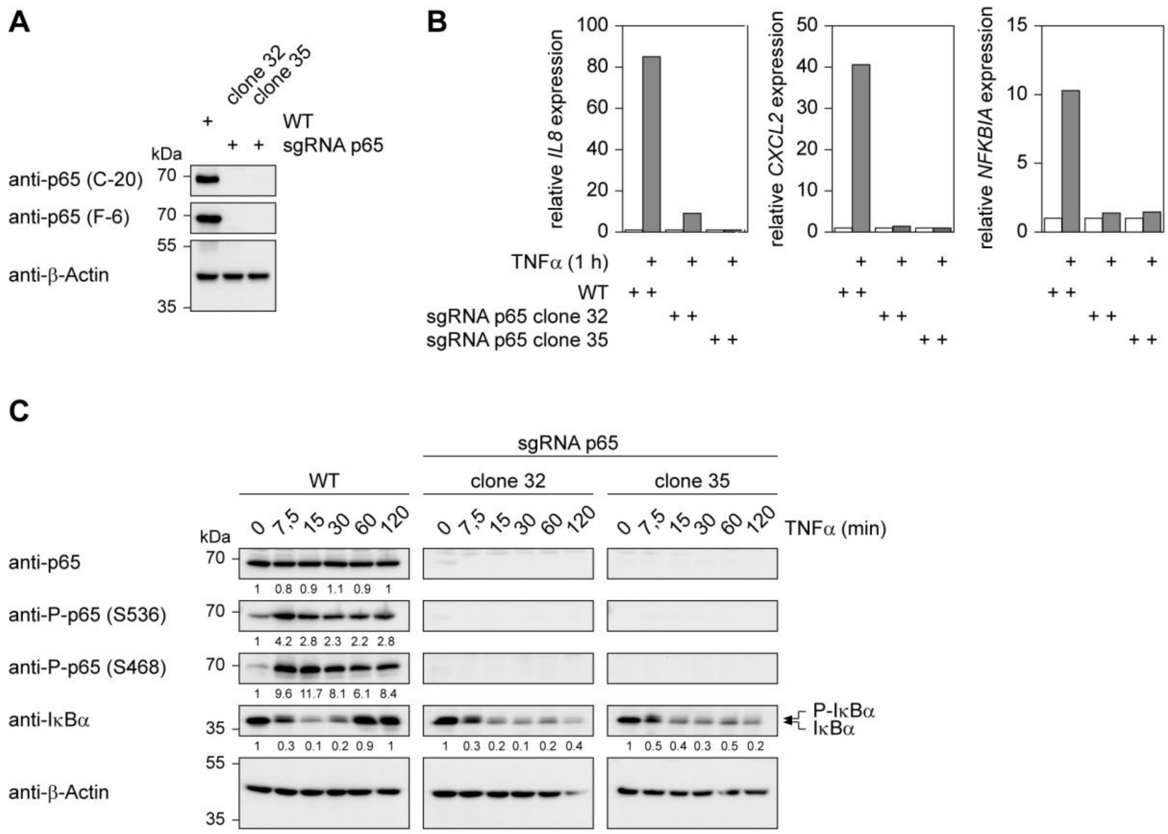

Figure S3. Characterization of p65-deficient HCT116 cells. (**A**) HCT116 WT cells and HCT116 cells where p65 was knocked out by CRISPR-Cas9 were lysed. Lysates containing equal amounts of protein were used for western blot analysis to detect p65 as revealed by antibodies recognizing the N-terminus (F-6) and C-terminus (C-20) of the protein, β-Actin was used as a loading control. (**B**) HCT116 WT and p65 knockout cells were stimulated for 1 h with TNF*α* (20 ng/mL), followed by extraction of RNA and analysis of *IL8*, *CXCL2* and *NFKBIA* expression by RT-qPCR. One representative experiment is shown. (**C**) HCT116 WT and p65 knockout cells were stimulated for the indicated periods with TNF*α* (20 ng/mL). Cell lysates were prepared and analyzed by western blotting for the occurrence and phosphorylation of the indicated proteins. Normalized intensity ratios are given for each band, the intensity of the DMSO-treated control was set as 1. β-Actin was used as housekeeping protein to ensure equal protein loading, one out of three experiments is shown.

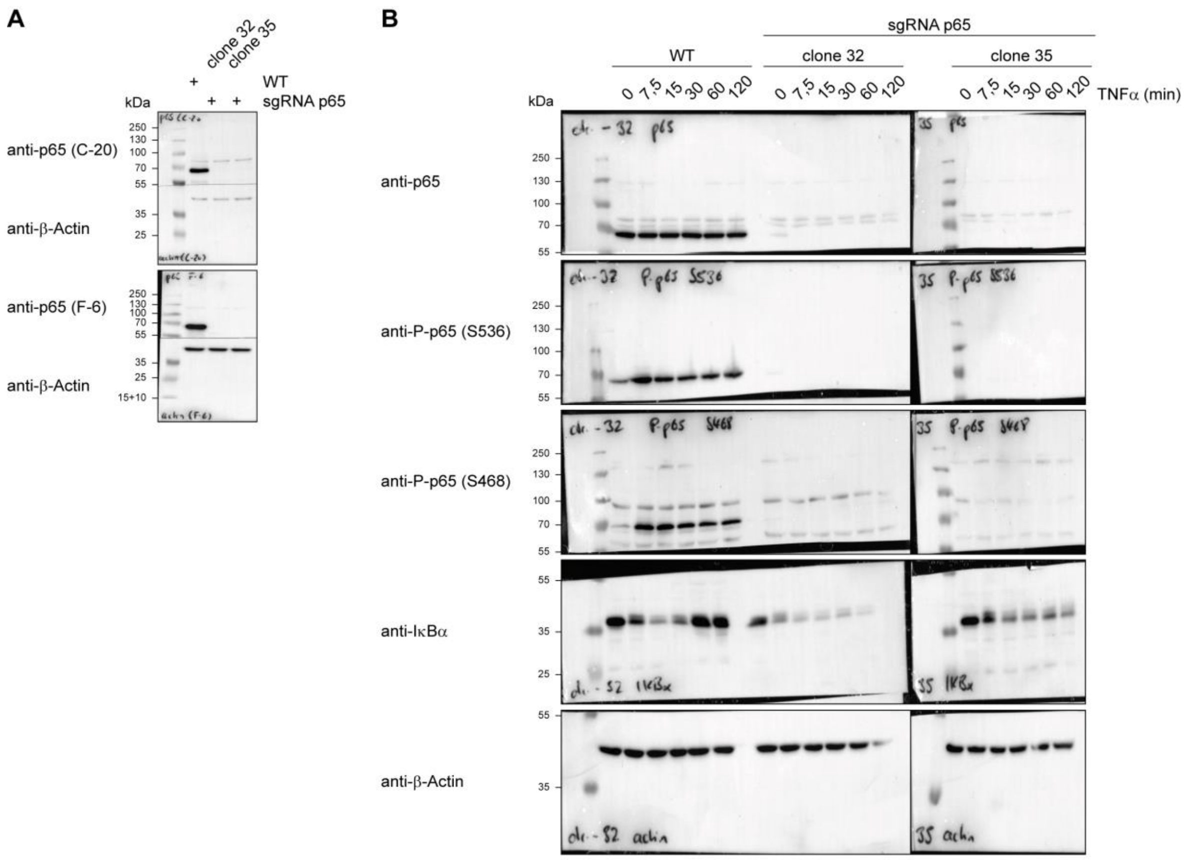

Figure S4. Blots from Figure S3 A and C.

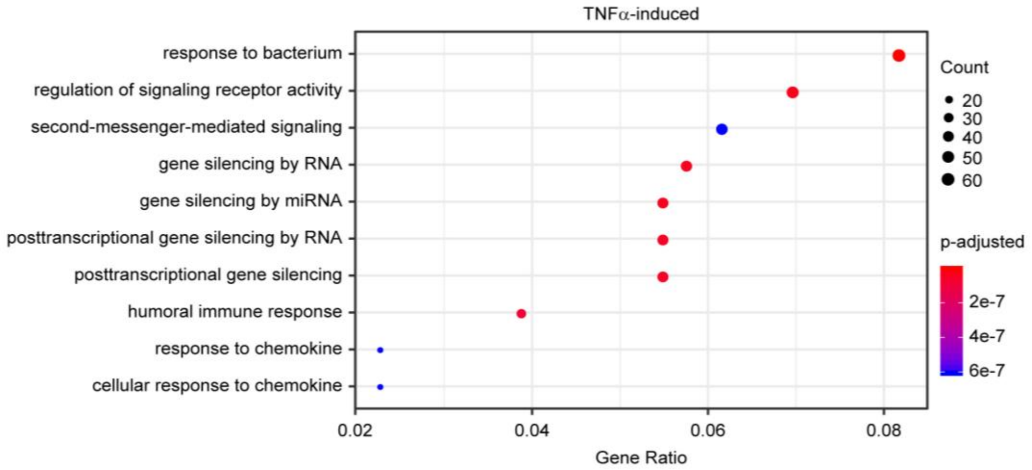

Figure S5. GO analysis of TNFα-induced genes. TNFα-induced genes were analyzed for overrepresentation of the GO term "biological process". Significance of enrichment is indicated by adjusted *p*-values and dot colors. Dot size shows the relative number of proteins in the experimental set compared to all proteins belonging to the respective GO term.

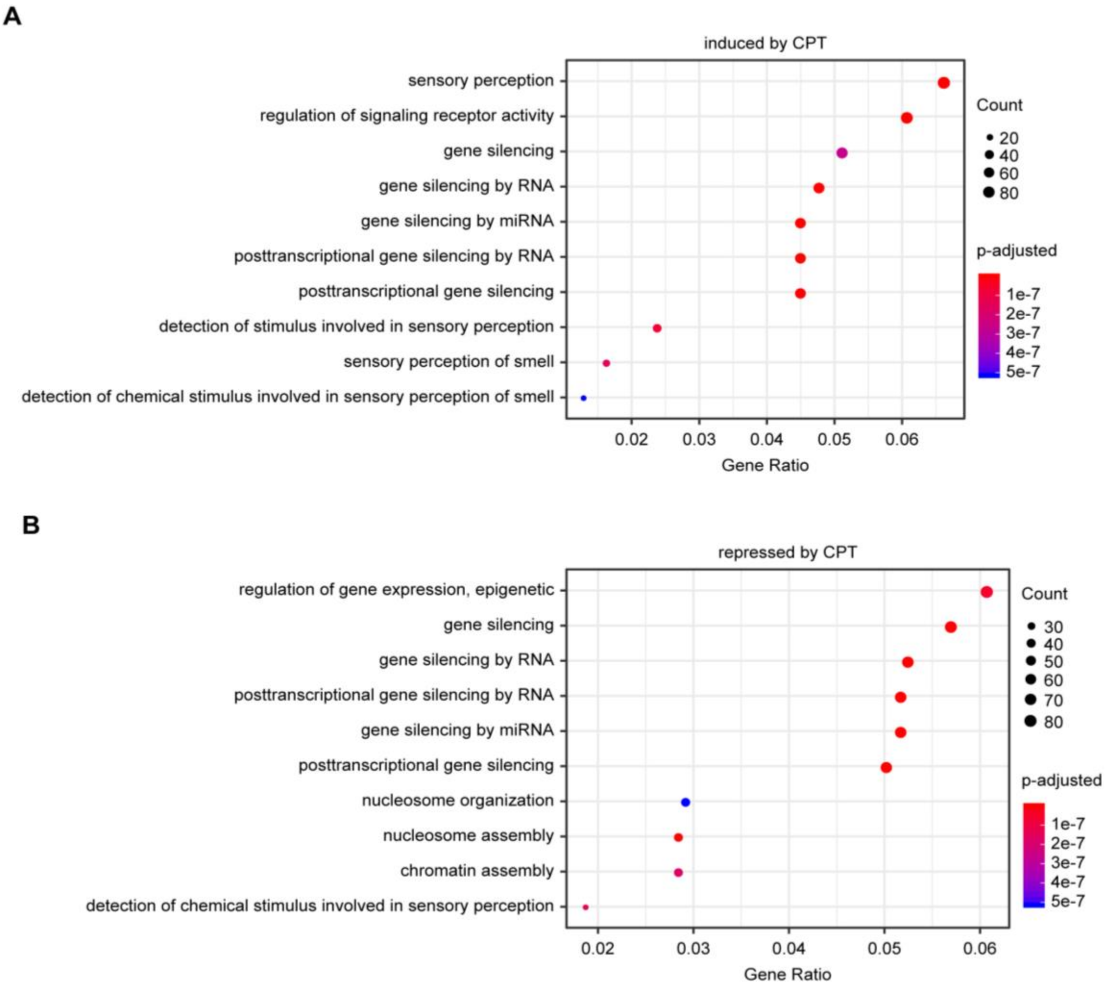

Figure S6. GO analysis of upregulated and downregulated genes in response to CPT under basal conditions. Genes either induced (**A**) or repressed (**B**) by CTP were analyzed for overrepresentation of the GO term "biological process". Significance of enrichment is indicated by adjusted *p*-values and dot colors. Dot size shows the relative number of proteins in the experimental set compared to all proteins belonging to the respective GO term.

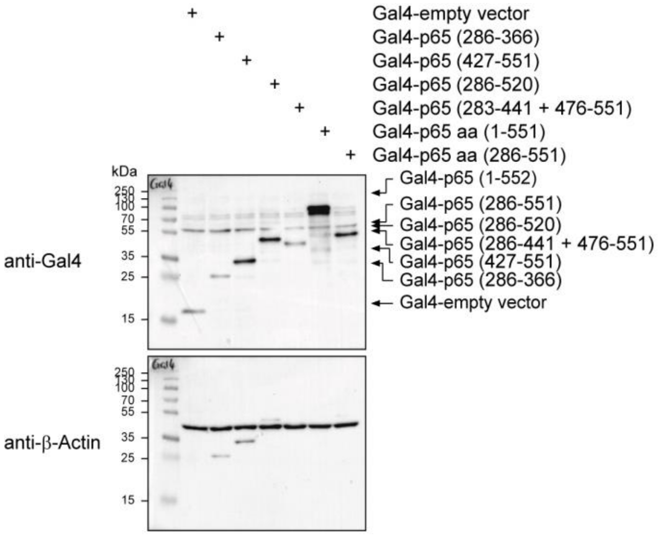

Figure S7. Blots from Figure 3C.

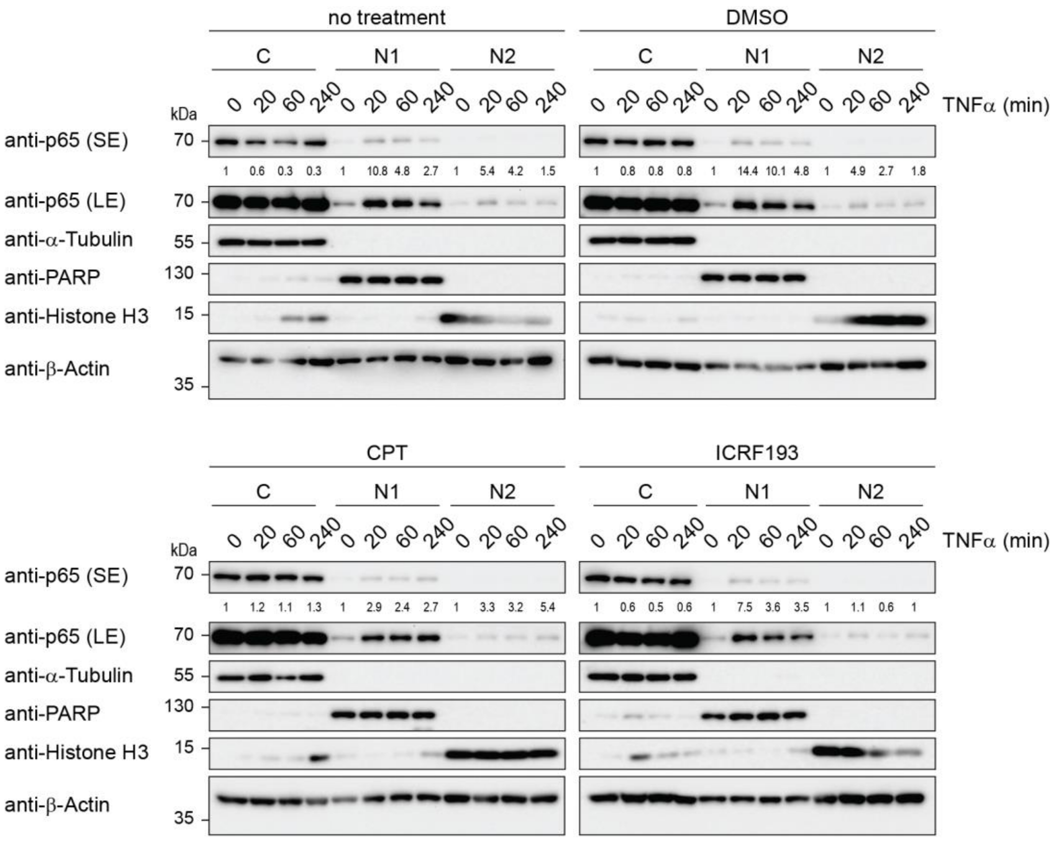

Figure S8. Effects of TOP1 and TOP2 inhibitors on nuclear translocation of NF-κB p65. HCT 116 cells were treated with TOP inhibitors and TNFα for the indicated periods and cells were fractionated into the cytosolic (C), soluble nuclear (N1) and insoluble nuclear (N2) fractions. These fractions were analyzed for the kinetics of p65 nuclear import by immunoblotting. The purity of the fractions was controlled by blotting for tubulin (C), PARP (N1) and histone H3 (N2). Short exposures (SE) and long exposures (LE) are shown for p65. Normalized intensity ratios are given for p65, the intensity of p65 and β-Actin at the time-point 0 min TNFα was set as 1 for each fraction.

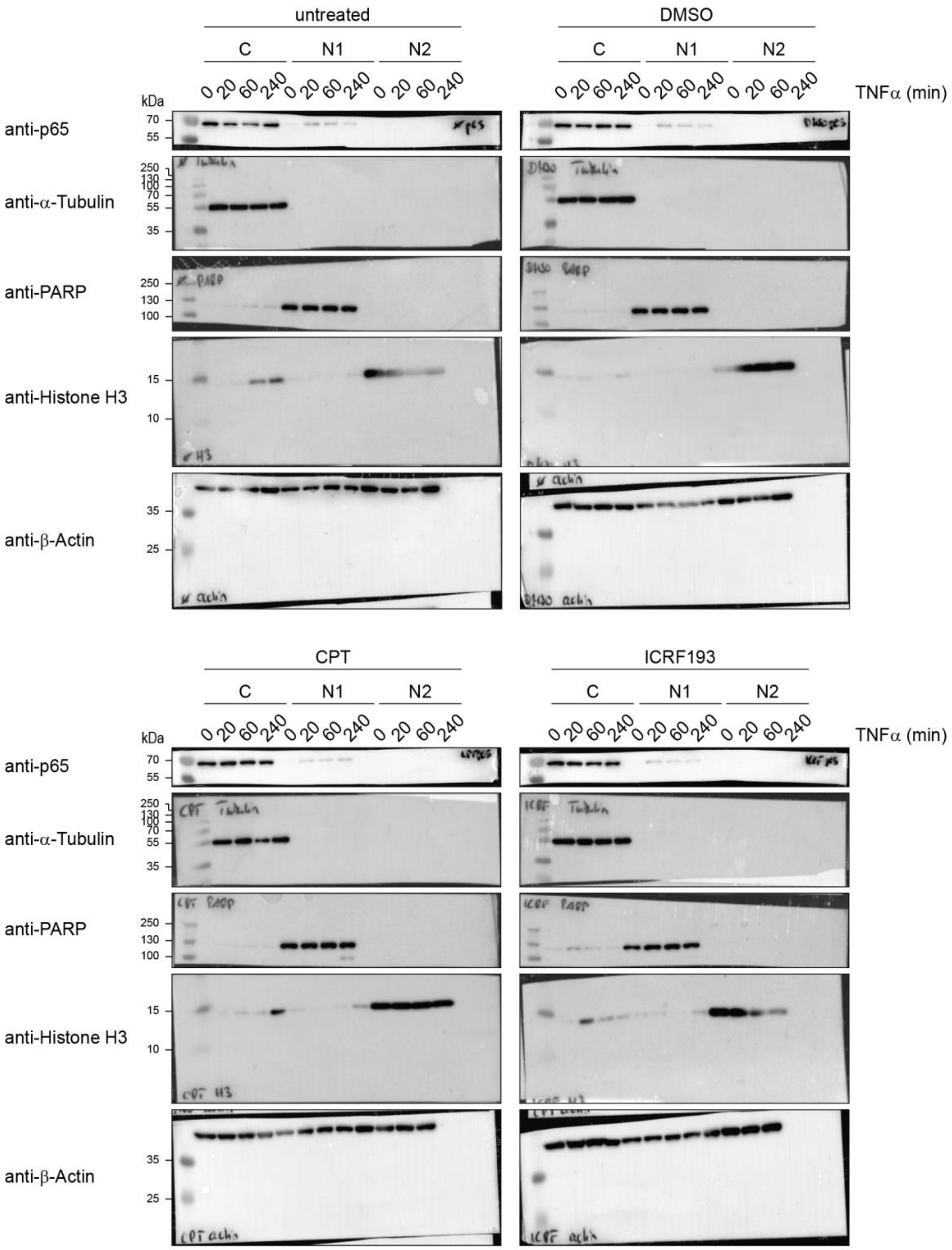

Figure S9. Blots from Figure S8.

Table S1. List of all genes with a log_2 fold change >1 upon 1 h TNFα-stimulation and ≥20 RNA-seq reads in at least one condition detected in samples from HCT116 cells. Log_2 fold change-values are given for each condition and gene. sorted in descending order starting with the highest log_2 fold change upon 1 h TNFα-stimulation in HCT116 control cells.

Gene	Control			sgRNA p65		
	1 h TNFα	1 h TNFα 2 h 5 µM CPT	1 h TNFα 2 h 5 µM ICRF193	1 h TNFα	1 h TNFα 2 h 5 µM CPT	1 h TNFα 2 h 5 µM ICRF193
CXCL8	6.42	2.61	5.61	1.28	−0.42	3.44
CXCL1	6.23	2.23	4.69	0.05	−0.25	−1.00
CSF2	5.73	0.55	7.30	−0.05	−1.42	−3.44
CXCL2	4.47	2.07	4.63	0.28	−0.65	−1.58
BIRC3	4.13	0.49	4.37	0.85	0.02	0.05
CXCL3	3.89	1.77	4.08	−1.05	−0.91	1.00
TNFAIP3	3.55	1.78	3.13	0.26	0.24	0.12
NFKBIA	3.27	0.90	3.10	0.12	−0.04	0.31
NUAK2	3.02	1.28	3.12	−0.32	−0.89	−0.18
NFKBIZ	2.90	0.53	2.73	−0.10	0.18	0.47
JUN	2.47	0.65	2.85	2.71	0.77	2.56
CD83	2.04	0.13	2.03	−0.08	0.10	0.09
IRF1	2.01	0.66	1.88	0.06	0.08	0.32
C10orf55	1.99	1.00	1.74	−0.18	−0.14	0.00
EDN1	1.71	−0.96	1.39	1.28	−0.65	0.59
JUNB	1.61	0.44	1.59	0.02	−0.05	0.17
EFNA1	1.52	−0.05	1.72	−0.39	−0.27	0.10
TNFAIP2	1.49	0.22	1.48	−0.15	−0.12	0.04
BCL3	1.45	−0.06	1.01	0.14	−0.19	−0.50
MAP3K8	1.43	−0.08	1.64	−0.89	0.09	−0.83
ZFP36	1.34	0.20	1.26	0.10	0.30	0.19
ELF3	1.29	0.30	1.11	0.00	−0.15	0.17
N4BP3	1.28	0.63	1.12	−0.32	−0.13	−0.15
ZC3H12A	1.28	0.32	1.06	0.14	−0.15	0.63
MIR3916	1.26	−0.51	−0.01	0.12	−0.48	0.15
NFKB2	1.26	0.57	1.26	0.02	−0.04	0.35
LYRM5	1.14	−0.72	0.42	0.36	1.40	0.30
PHOSPHO1	1.10	−0.08	−0.80	−0.99	0.47	1.10
STX11	1.10	0.08	1.46	0.28	−0.16	0.59
IKBKE	1.09	0.29	1.03	−0.17	−0.16	0.17
ATF3	1.07	0.37	0.98	1.06	−0.23	0.94
CFLAR-AS1	1.06	0.61	0.24	0.75	−0.57	−0.68
C18orf32	1.04	−0.15	0.29	−0.89	−0.42	−0.26
CSF1	1.03	−0.38	1.11	0.28	−0.89	−0.11
COX20	1.03	0.38	−0.22	0.08	0.10	−0.15
KLF10	1.02	0.10	1.24	−0.12	−0.15	−0.22
IER3	1.02	0.40	0.99	0.15	−0.06	0.23
GUCY1B2	1.01	0.73	−0.14	0.12	0.90	0.00
RELB	1.01	0.33	1.02	−0.42	−0.15	0.38
WNT10A	1.00	0.28	0.98	0.13	0.06	0.19

Table S2. Statistical analysis of RNA-seq experiments.

Sample	Number of Input Reads	Uniquely Mapped Reads Number	Uniquely Mapped Reads %
WT	40457047	28692789	70.92
WT + CPT	33542381	23184038	69.12
WT + ICRF193	29938841	21263022	71.02
WT + TNF	32444181	22837367	70.39
WT + TNF + CPT	39300078	26812397	68.22
WT + TNF + ICRF193	33219716	23448789	70.59
p65-/-	37749910	27014424	71.56
p65-/- + CPT	37051013	25987161	70.14
p65-/- + ICRF193	15796987	11738598	74.31
p65-/- + TNF	14879528	11065827	74.37
p65-/- + TNF + CPT	15788867	11381788	72.09
p65-/- + TNF + ICRF193	15987496	11919596	74.56

Table S3. Antibodies, plasmids and reagents.

Antibodies

Primary Antibody (Clone)	Species	Supplier
p65 (C-20)	rabbit pAb	Santa Cruz #sc-372
p65 (F-6)	mouse mAb	Santa Cruz #sc-8008
p65 phospho serine 536 (9BH1)	rabbit mAb	Cell Signalling #3033
p65 phospho serine 468	rabbit pAb	Cell Signalling #3039
IκBα (C21)	rabbit pAb	Santa Cruz #sc-371
TOP1	rabbit pAb	Bethyl #A302-589-A
histone H3	rabbit pAb	Abcam #ab1791
histone H3 acetyl lysine 9	rabbit pAb	Millipore #07-352
histone H3 acetyl lysine 27	rabbit pAb	Diagenode #C15410174
Pol II (N-20)	rabbit pAb	SCBT #sc-899
Pol II phospho serine 2	rabbit pAb	Abcam #ab5095
Pol II phospho serine 5	rabbit pAb	Abcam #ab5131
Gal4	rabbit	M. L. Schmitz
β-Actin	rabbit pAb	Abcam #ab8227
normal rabbit IgG	rabbit	Cell Signalling #2729S
**Secondary Antibody**	**Conjugated to**	**Supplier**
goat-anti-rabbit IgG	HRP	Dianova #111-035-144
goat-anti-mouse IgG	HRP	Dianova #112-035-143

Plasmids

Plasmid	Origin	Reference
pX459	F. Zhang	PMID: 24157548
pX459-sgRNA hu p65	M.L. Schmitz	PMID: 30526044
pCI-*Renilla* luc	M.L. Schmitz	PMID: 28615693
Gal4-empty vector	M.L. Schmitz	PMID: 1935902
Gal4-p65 (1-551)	M.L. Schmitz	PMID: 1935902
Gal4-p65 (286-366)	M.L. Schmitz	PMID: 7797554
Gal4-p65 (427-551)	M.L. Schmitz	PMID: 7797554
Gal4-p65 (286-520)	M.L. Schmitz	PMID: 7797554
Gal4-p65 (286-441 + 476-551)	M.L. Schmitz	PMID: 7797554
Gal4-p65 (286-551)	M.L. Schmitz	PMID: 1935902

DNA-Oligonucleotides for CRISPR/Cas9-mediated p65 knock-out

Oligo Name	Sequence (5′ to 3′)
px459-hu p65-for	CACCGCTTCCGCTACAAGTGCGA
px459-hu p65-rev	AAACTCGCACTTGTAGCGGAAGC

DNA-Oligonucleotides for qPCR

Oligo Name	Sequence (5′ to 3′)
hu CXCL2-for	AGCTTGTCTCAACCCCGCATC
hu CXCL2-rev	GGGCAGGGCCTCCTTCAGG
hu IL8-for	AGTGGACCACACTGCGCCAA
hu IL8-rev	TCTCCACAACCCTCTGCACC
hu NFKBIA-for	CCCTACACCTTGCCTGTGAG
hu NFKBIA-rev	CACCAAAAGCTCCACGATGC
hu CXCL10-for	GCAGAGGAACCTCCAGCTTCAGCA
hu CXCL10-rev	TGCTGATGCAGGTACAGCGTACAG
hu TNAFIP3-for	CACGCTCAAGGAAACAGACA
hu TNFAIP3-rev	CATGGGTGTGTCTGTGGAAG
hu ICAM1-for	AGCTTCGTGTCCTGTATGGC
hu ICAM1-rev	TTTTCTGGCCACGTCCAGTT
hu TPI-for	GGACTCGGAGTAATCGCCTG
hu TPI-rev	TGTTGGGGTGTTGCAGTCTT
hu Ccl5-for	CGTGCCCACATCAAGGAGTA
hu Ccl5-rev	TCTTCTCTGGGTTGGCACAC
hu CSF1-for	GGAGTCTGTCTTCCACCTGC
hu CSF1-rev	GAATCCGCTCTCTGAGGCTC
hu LIF-for	GTGCAGCCCATAATGAAGGT
hu LIF-rev	CCCCTGGGCTGTGTAATAGA
hu Ccl2-for	ATCAATGCCCCAGTCACCTG
hu Ccl2-rev	TCTCCTTGGCCACAATGGTC

DNA-Oligonucleotides for ChIP-qPCR

Oligo Name	Sequence (5′ to 3′)	Amplicon Length
enhancer flanking 1-for	AAGGAACTTTTCTTCCCACGA	172 bp
enhancer flanking 1-rev	GCCATTAGGAGCCACAAAAT	
enhancer-for	AAAGGGGATTCAAAGGGAGA	182 bp
enhancer-rev	CTTCTCCAGGCTCCATTCAG	
enhancer flanking 2-for	TACACAGAGGTCACCGTCCA	212 bp
enhancer flanking 2-rev	GGTTGCTGAAACACAGCTCA	
control-for	GGTGGGAGGGAGGTGTTATCTAATG	136 bp
control-rev	ATCATGGGTCCTCAGAGGTCAGAC	
promoter-for	AAGAAAACTTTCGTCATACTCCG	170 bp
promoter-rev	TGGCTTTTTATATCATCACCCTAC	
exon 1-for	TGCATAAGTTCTCTAGTAGGGTGATG	302 bp
exon 1-rev	TGTTTGTTACCAAAGCATCAAGA	
exon 2-for	AAGGAAGTAGCTGGCAGAGC	200 bp
exon 2-rev	AATTTCTGTGTTGGCGCAGT	
intron 1-for	TGCTTTGGTAACAAACATCCTTT	306 bp
intron 1-rev	CAAATCTGAGGCTTGTCAATG	
intron 3-for	ACCATACATAGTTTGCCCAGGA	199 bp
intron 3-rev	GAAGGAAAGTAGGGTTCTTGAAAA	

Reagents

Name	Working Concentration	Supplier
human TNFα	20 ng/mL	ImmunoTools #11343015
human IL-1	10 ng/mL	Dr. M. Kracht
Dimethyl sulfoxide		Sigma Aldrich #41639
Camptothecin	0.5–10 µM	Tocris #1100
ICRF193	0.5–10 µM	Enzo life sciences #BML-GR332-0001
Topotecan	5 µM	Tocris #4562
SN-38	0.5-10 µM	Cayman Chemical #15632
Teniposide	5 µM	Cayman Chemical #14425
Etoposide	5 µM	Biotrend # BG0186
Amsacrine	5 µM	Cayman # 22223

© 2019 by the authors. Licensee MDPI, Basel, Switzerland. This article is an open access article distributed under the terms and conditions of the Creative Commons Attribution (CC BY) license (http://creativecommons.org/licenses/by/4.0/).
